# Label-free proteomics uncovers SMC1A expression is Down-regulated in AUB-E

**DOI:** 10.1186/s12958-021-00713-4

**Published:** 2021-03-02

**Authors:** Yingxian Jia, Jie Luo, Yibing Lan, Chunming Li, Linjuan Ma, Xiaoming Zhu, Fei Ruan, Jianhong Zhou

**Affiliations:** Women’s Hospital, School of Medicine, Zhejiang University, Zhejiang, China

**Keywords:** Abnormal uterine bleeding, Human endometrium, Proteomic analysis, Primary endometrial disorder

## Abstract

**Background:**

While heavy menstrual bleeding (HMB) is a prevalent symptom among women with abnormal uterine bleeding caused by endometrial disorder (AUB-E) seeking gynecologic care, the primary endometrial disorder remains poorly understood.

**Methods:**

Five human endometrial samples from women with AUB-E and the age-matched healthy women were selected, respectively. Proteins from the samples were analyzed by a linear ion trap (LTQ)-Orbitrap Elite mass spectrometer based label-free proteomic approach. The purpose protein was validated by western blot and immunohistochemistry staining.

**Results:**

A total of 2353 protein groups were quantified under highly stringent criteria with a false discovery rate of < 1% for protein groups, and 291 differentially expressed proteins were significantly changed between the two groups. The results showed that the down-regulation of structural maintenance of chromosomes protein 1A (SMC1A) in AUB-E patients. Next, this change in the glandular epithelial cells was validated by immunohistochemistry.

**Conclusion:**

The results indicated a novel mechanism for the cause of AUB-E, as down-expression SMC1A potentially regulated the cell cycle progression in endometrial glandular epithelium further led to bleeding.

**Supplementary Information:**

The online version contains supplementary material available at 10.1186/s12958-021-00713-4.

## Background

Abnormal uterine bleeding (AUB) is one of the most common gynecological diseases in women, accounting for 33% of patients attending the gynecology clinic and two-third cases of hysterectomy [1]. To meeting the requirement of standardized definitions and terminologies, using the acronyms of underlying causes and the mechanisms involved in the genesis of AUB, the “PALM-COEIN” classification system as a suitable system for widespread international use since 2011 [2], which has been accepted by the International Federation of Gynecology and Obstetrics (FIGO) [3, 4]. In this system, the COEI group is involved in entities not defined by imaging or histopathology (“non-structural”). AUB-E that occurs in the context of a structurally normal uterus with regular menstrual cycles without evidence of coagulopathy is likely to have an underlying endometrial cause, primary endometrial disorder [4]. This condition is often characterized by heavy menstrual bleeding (HMB), usually at fairly regular intervals and with a pattern of daily menstrual loss similar to normal menses, with ~ 90% of the flow occurring during the first 3 days [5]. Although AUB-E may be implicated in many women, a lack of clinically available specific tests or biomarkers means that practical testing for such disorders is not yet feasible [6], which are still has neglected, inadequate diagnosis in AUB-E patients. It occurs in 9–14% of women of all ages and is responsible for about 25% of gynecological surgeries which results from a universally applicable approach deficiency in medical therapy [7, 8]. However, a well-structured history and examination often help, there is no commercially available testing. Hence, a clear role in understanding the etiology of AUB-E and developing biomarkers.

Accompanied by rapid developments and improvements in the field of mass spectrometry (MS), proteomics has employed for discovering biomarkers and answering multidisciplinary scientific questions in different aspects of medicine, such as the elucidation of pathways affected in disease or prediction for the high-risk individuals of developing the disease [9]. Expression analysis directly at the protein level is necessary to unravel the critical changes that occur as part of disease pathogenesis [10]. Therefore, proteomics provides extremely useful tools for investigating the etiology of patients with AUB-E. So far, however, proteomic analysis has been used in other gynecological diseases, there has not been applied to compare the differential protein expression profile for investigating the etiology of AUB-E [11].

**Fig. 1:**
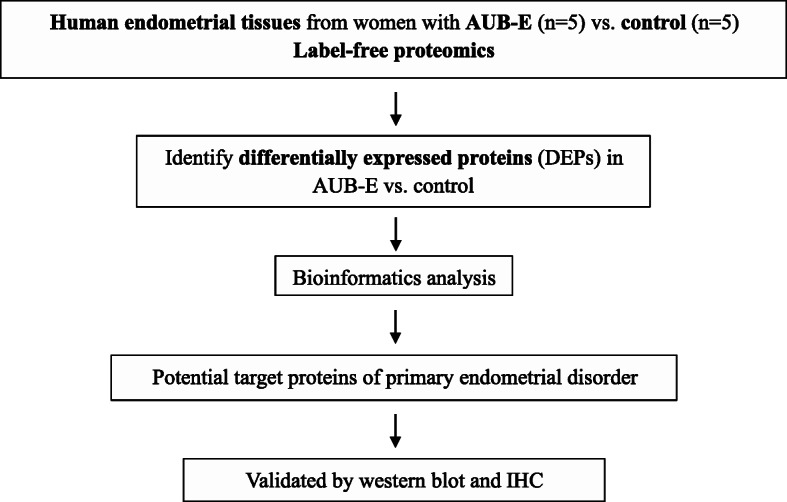
Experimental design and the workflow in this study. The human endometrial tissues in the proliferative phases between AUB-E patients (group E) and endometrial healthy women from the control group who without AUB (group C) were carried out a label-free proteomic analysis for investigating proteome changes. Identified potential proteins were validated by western blot and immunohistochemistry

In this study, the human endometrial tissues in the proliferative phases between AUB-E patients (group E) and endometrial healthy women from the control group who without AUB (group C) were carried out the label-free proteomic analysis and identified biomarkers of relevance to the endometrial disorder after validation. This study will be likely to provide a reference for further study on the pathogenesis of AUB-E. The workflow of this study was shown in Fig. 1.

## Material and methods

### Patients and control subjects

This study was carried out at the Department of Gynecology in Women’s Hospital, School of Medicine, Zhejiang University. The selection criteria included an examination to rule out any other known cause of AUB, thus strictly adhering to the definition of AUB-E. All non-pregnant females completed a face-to-face interview to obtain their detailed histories, including demographic characteristics, menstrual and medical history combining pregnancy test, routine hematological and coagulation studies, and transvaginal ultrasound for the exclusion of other possible causes of AUB. The selection criteria were permanent residence in Zhejiang Province, aged between 20 and 40 years with self-reported HMB assessed by the Pictorial Blood Assessment Chart (PBAC) [12]. Any female presenting with systemic diseases like thyroid dysfunction, diabetes mellitus, hypertension, chronic liver, kidney disease and organic genital tract lesion, uterine and ovarian tumors, pregnancy-related causes, intrauterine contraceptive devices, endometriosis, and pelvic inflammatory disease were excluded. Endometrial tissues were obtained by curettage in the proliferative phases of the menstrual cycle (days 7–10; *n* = 5) and examined thoroughly to note the histopathological details and classified into group E.

Controls were selected from women of similar age group with normal menstrual history and no endometrial abnormalities undergoing curettage following laparoscopic sterilization (*n* = 3), and assessment of tubal patency (*n* = 2). The proliferative endometrial biopsies were carried out with a histopathology diagnosis to exclude leiomyoma, endometriosis, polyp, endometrial inflammatory and malignancy. All women had not received steroid hormone therapy in the last 6 months.

### Sample preparation

All endometrial tissues (~ 20 mg) from group E (age range 26–37, median age 28) and group C (age range 27–34, median age 28) were transported immediately to the laboratory in phosphate-buffered saline (PBS) on ice. Samples were extensively washed with PBS to remove any blood and were distributed into two parts. One part was sent for histopathology diagnosis, and another was stored at − 80 °C for further proteomic or western blot analysis. The total duration from endometrial biopsy removal to sample freezing was controlled to be 15 min.

### Protein digestion using FASP method

Snap-frozen endometrial tissues were added to SDS lysis buffer (2% SDS, 0.1 M DTT, 0.1 M Tris-HCl, pH 7.6), and homogenized. Following centrifugation (16,000 *g* × 5 min at 4 °C), the supernatant was transferred to a fresh tube and the pellet re-extracted as above. The mixture was incubated in boiling water for another 5 min and sonicated for 20 min and centrifuged at 16000 *g* × 30 min at 20 °C. The supernatants were collected and determined protein concentration by a NanoDrop® ND-1000 Spectrophotometer.

200 μg of the sample was digested by the FASP procedure as described [13]. Each sample was concentrated at 14000 *g* × 40 min at 20 °C in 30 k Microcon filtration devices (Millipore, USA). Then, 200 μL of urea buffer (8 M urea, 0.1 M Tris-HCl, pH 8.5) was added to the sample and centrifuged again for another 14 min. This step was repeated one more time. The concentrate was mixed with 100 μL of 50 mM iodoacetamide (IAA) in urea buffer and incubated for an additional 40 min at room temperature in darkness. Following centrifugation at 14000 g × 15 min, the sample was diluted with 200 μL of urea buffer and centrifuged two more times. Then, 200 μL of 50 mM NH4HCO3 was added and centrifuged again. This step was repeated twice. Finally, 50 μL of 50 mM NH4HCO3 and trypsin (1:50) was added to the sample, which was then incubated at 37 °C overnight. Eluted peptides were collected by centrifugation followed by two washes with 40 μL of 50 mM NH4HCO3 and vacuum dried. Desalting was then carried out using ZipTip C18 (Millipore, USA) following the manufacturer’s instructions and the samples were vacuum dried. Finally, peptide digests were resuspended in 10% acetonitrile (ACN) in 0.1% formic acid (TFA) and detected the protein concentration.

### LC-MS/MS analysis

High-performance liquid chromatography (HPLC) was used for sample separation using the EASY-nLC 1000 (Thermo Fisher Scientific, USA) with a binary buffer system consisting of 0.1% TFA in water (buffer A) and ACN in 0.1% TFA (buffer B). Samples were loaded into the precolumns (20 mm × 75 μm, 3 μm-C18, Thermo scientific EASY column, USA) using an auto-sampler and separated by analytical columns (150 mm × 50 μm, 2 μm-C18, Thermo scientific EASY column, USA) at a flow rate of 10 μL/min. The liquid phase gradients were as follows: 3–8% buffer B for 0–10 min, 8–20% buffer B for 10–120 min, 20–30% buffer B for 120–137 min, 30–90% buffer B for 137–143 min, and 90% buffer B for 143–150 min. The separated peptides were then analyzed by a linear ion trap (LTQ)-Orbitrap Elite mass spectrometer (Thermo Fisher Scientific, USA) fitted with an electrospray ionization (ESI) source. In the positive ion mode, a full scan range from *m/z* 300 to 2000 with a resolution of 60,000 (200 *m/z*). The top 20 precursors of the highest abundance in the full scan were selected and fragmented by collision-induced dissociation (CID) function and analyzed in MS/MS, where a resolution was 15,000, a normalized collision energy was set as 35%. The following dynamic exclusion settings were also used: repeat counts 1; repeat duration 30 s; exclusion duration 60 s. Data were post-processed using the QualBrowser part of Thermo Scientific Xcalibur 2.2 software.

### Data analysis

Unprocessed raw files were searched against the UniProtKB *Homo sapiens* database comprised of 188,386 sequences (www.uniprot.org) by the search engines: PEAKS® Studio 8.0 (Thermo Fisher Scientific). The search parameters are set as follows: mass tolerance for precursor ion was 10 ppm and mass tolerance for production was 0.02 Da. Carbamidomethyl (C) was specified as fixed modifications, Oxidation (M) was specified as dynamic modification, and acetylation was specified as N-terminal modification in PEAKS® Studio 8.0. A maximum of 2 missed cleavage sites was allowed. To improve the quality of analysis results, the software further filtered the retrieval results: Combining the identified PSMs (with the credibility of more than 99%) and protein (contained at least 1 unique peptide) were retained and performed with a false discovery rate (FDR) no more than 1.0%. The protein quantitation results were statistically analyzed by the *t*-test. The proteins whose quantitation significantly different between AUB-E and control groups (*P* ≤ 0.05 and fold change (FC) ≥ 1.5) were defined as differentially expressed proteins (DEPs). The principal components analysis (PCA) of all samples was also checked.

### The functional analysis of DEPs

DEPs were used for volcanic map analysis, cluster heat map analysis, and enrichment analysis of Kyoto Encyclopedia of Genes and Genomes (KEGG) [14]. The protein-protein interactions (PPI) of probable pathways from enrichment analysis was predicted using Cytoscape software (Version. 3.8.0, https://cytoscape.org/) [15].

### Western blotting

Western blot was used to validate the differential expression of structural maintenance of chromosomes protein 1A (SMC1A) between group C and E. In brief, each tissue was homogenized for 10 min in RIPA buffer and protease inhibitor cocktail. Then, the crude extract was sonicated for 1 min and centrifuged at 10000 g × 10 min at 4 °C. The supernatant was detected the protein concentration and denatured at 95 °C for 10 min. After that, 10 μl of sample ran on a 12% SDS-PAGE, transferred onto polyvinylidene fluoride (PVDF) membranes, blocked with 5% bovine serum albumin (BSA) for 1 h at room temperature, and incubated in anti-SMC1A (ab243875, Abcam, Cambridge, UK) antibodies 1:1000 diluted overnight at 4 °C. After washing with tris buffered saline with Tween 20 (TBST), membranes were incubated with anti-rabbit IgG horseradish peroxidase-labeled antibody produced in goat (ab6721, Abcam, Cambridge, UK) for 1 h; then, the fluorescence images were acquired by Super ECL Detection Reagent. Each gel was loaded with a ladder from 5 to 250 kDa for indicating the molecular weight of the corresponding gel band. GAPDH protein was used as a loading control to normalize the western blot data, and the normalized abundance of SMC1A is calculated by dividing the band intensity by the corresponding GAPDH band intensity.

### Immunohistochemistry

Other samples were retrieved through the electronic medical system for immunohistochemical (IHC) analysis of archived samples. Routine histological examination using hematoxylin and eosin staining was done to identify any abnormalities.

For IHC analysis, endometrial tissue samples were paraffin-embedded, cut into 5-μm-thick sections to identify the localization of SMC1A (ab243875, Abcam) in the endometrium. All subsequent incubations were performed in a humidified chamber. Slides were first blocked with 10% goat serum (Invitrogen, Eugene) for 30 min at room temperature; then, they were incubated overnight at 4 °C with a rabbit anti-SMC1A antibody (1:200 dilution). After washing, the samples were incubated with secondary antibody, goat-anti-rabbit (Invitrogen; 1:200 dilution) for 1 h. Slides were washed and exposed to diaminobenzidine (DAB, Sigma) to counterstain the nucleus. Finally, the stained sections were scanned by the Grundium Ocus® scanner (Grundium, Finland).

For each sample, protein expression intensities were determined in the glandular epithelium and the stroma area using Image J software (National Institutes of Health, Bethesda, Maryland, USA), respectively. Briefly, the intensity of staining was graded as 0 (negative), 1 (weak), 2 (moderate), or 3 (strong), as illustrated previously [16]. Assessment of immunopositivity in cell nuclei was conducted using a histological score (H-score) as 1 x % weak + 2 x % moderate + 3 x % strong. The specimens were inspected by two independent observers, who were “blind” to experimental conditions.

### Statistical analysis

Data were performed using the *t*-tests for means of two groups and the chi-square test for the composition ratios. Continuous and categorical variables were described as mean ± standard deviation (SD) and the number and percentage of subjects, respectively. The Mann–Whitney U test was used if continuous variables were non-normally distributed, and shown as median (25th percentile, 75th percentile). The *t-*tests were used to assess SMC1A expression (H-scores) in the glandular epithelium and the stroma area between the two groups. *P* values<0.05 were considered statistically significant by the SPSS software (Chicago, IL, version 23.0) and graphed with GraphPad PRISM software (V6.0; GraphPad Software Inc., San Diego, CA).

## Results

### Baseline data analysis

Baseline data of all participants were collected and analyzed. There was no significant difference in demography and menstrual characteristic among the two groups (*P* > 0.05) in Table 1. Although there were no statistically significant differences in hematoglobin (Hb), hematocrit (HCT), and baseline FSH levels and duration of flow and menstrual cycle length (all *P* values > 0.05), the PBAC scores showed a significant difference between the two groups (*P* = 0.000) (see Table 2).

**Table 1:** The demographic characteristic of all subjects

Demographic characteristic	AUB-E	Control	***P*** value
	n = 5	***n*** = 5	
**Age, years** Median(P25, P75)	28 (26, 37)	28 (27, 34)	1.00^a^
**Education, n (%)**			0.421^b^
Above college	1 (20)	1 (20)	
Middle school	2 (40)	4 (80)	
others	2 (40)	0	
**Employed status, n (%)**	5(100)	5 (100)	1.00^b^
**Income, n (%)**			0.310^b^
≤ 5000 RMB	4 (80)	2 (40)	
>5000 RMB	1 (20)	3 (60)	
**No smoking, n (%)**	5 (100)	5 (100)	1.00^b^

n, the number of subjects

^a^*P* value for the Mann–Whitney U-test. ^b^*P* value for the chi-square test

**Table 2:** The clinical details of all subjects

Clinical details	AUB-E	Control	***P*** value
	*n* = 5	*n* = 5	
**Menarche, years** Mean (SD)	14 (1)	13 (1)	0.073^a^
**Parity** Median(P25, P75)	1 (1, 1.5)	1 (1, 1.5)	1.000^b^
**Height, cm** Mean (SD)	159.80 (1.79)	161.20 (2.05)	0.283^a^
**Weight, kg** Mean (SD)	54.80 (5.01)	55.80 (7.50)	0.810^a^
**Body-mass index, kg/m**^**2**^ Mean (SD)	21.49 (2.28)	21.46 (2.75)	0.989^a^
**Menstruation duration, days** Mean (SD)	5.2 (1.8)	5.8 (0.8)	0.516^a^
**Menstrual cycle length, days** Median(P25, P75)	30 (29, 31)	30 (29, 30)	0.841^b^
**PBAC score** Mean (SD)	151.2 (26.2)	61.8 (8.9)	0.000^a,^*
**Hematoglobin, g/L** Median(P25, P75)	130 (88.5, 134)	136 (129, 138)	0.151^b^
**HCT** Mean (SD)	0.36 (0.07)	0.4 (0.024)	0.227^a^
**Baseline FSH, U/L** Median(P25, P75)	5.9 (5.1, 8.6)	5.45 (4.88, 7.60)	0.690^b^

n, the number of subjects; PBAC, the Pictorial Blood Assessment Chart; HCT, hematocrit; *: *P* < 0.05, which denoted a significant difference between AUB-E and Controls

^a^*P* value for the *t*-tests. ^b^*P* value for the Mann–Whitney U-test

### Protein identification

**Fig. 2:**
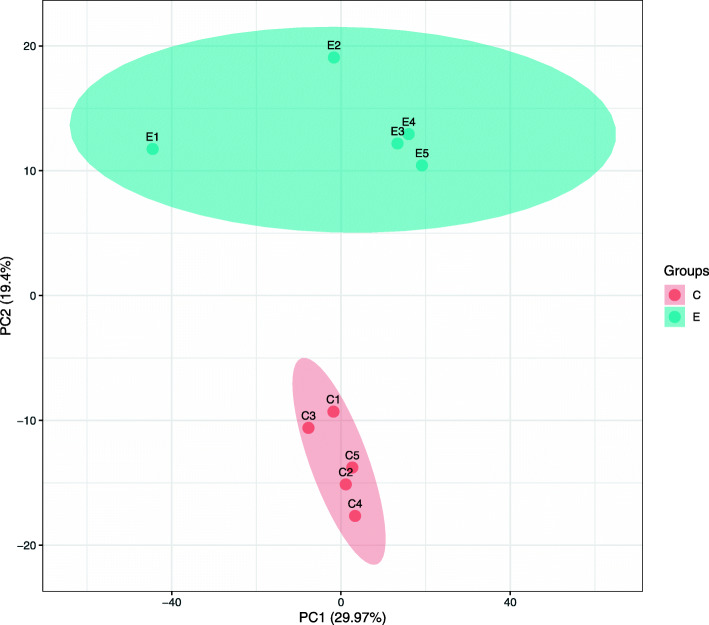
PCA analysis results between the AUB-E group and controls. E: each blue circle was represented a sample from group E, which comprised a blue block; C: each rad circle was represented a sample from group C, which comprised a red block

**Fig. 3:**
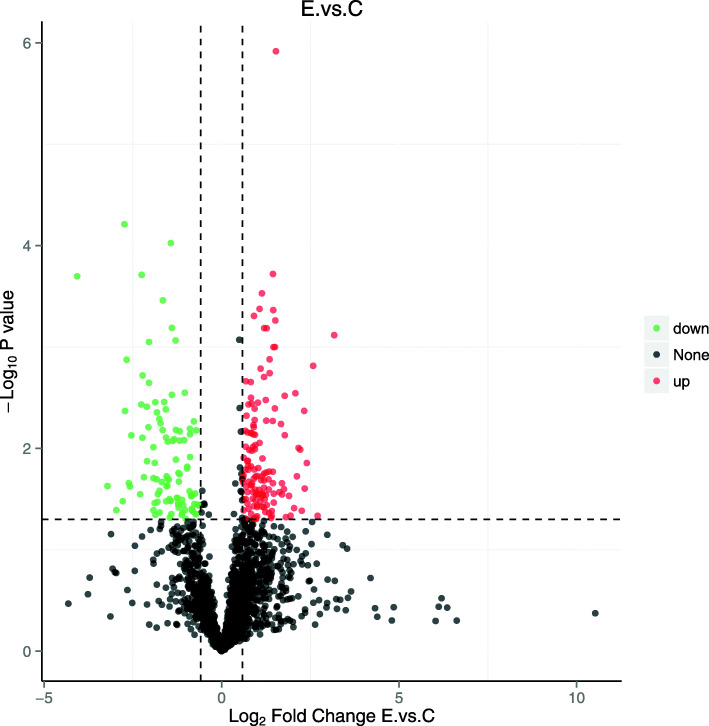
The protein-level volcano plot of quantified 1921 different proteins from the endometrial tissues. The logarithmic ratio of protein quantitative intensities in the E/C groups was plotted against negative logarithmic *p* values of the *t*-test performed from five replicates. E: the endometrial tissue from group E; C: the endometrial tissue from group C; Up: up-regulated proteins in red (FC ≥ 1.50, *P* < 0.05); Down: down-regulated proteins in green (FC ≤ 0.67, *P* < 0.05)

The experiment detected 2353 protein groups and quantified 1921 different proteins (Supplementary Table S1 and Table S2). The further screening revealed 291 DEPs in AUB-E patients compared with healthy controls, including 140 up-regulated proteins (FC ≥ 1.50, *P* < 0.05) and 151 down-regulated proteins (FC ≤ 0.67, *P* < 0.05) (Supplementary Table S3), and these are presented in heat map format in Supplementary Fig. S1. Moreover, PCA analysis and protein-level volcano plots were indicated in Figs. 2 and 3.

### KEGG pathway enrichment analysis of DEPs

**Fig. 4:**
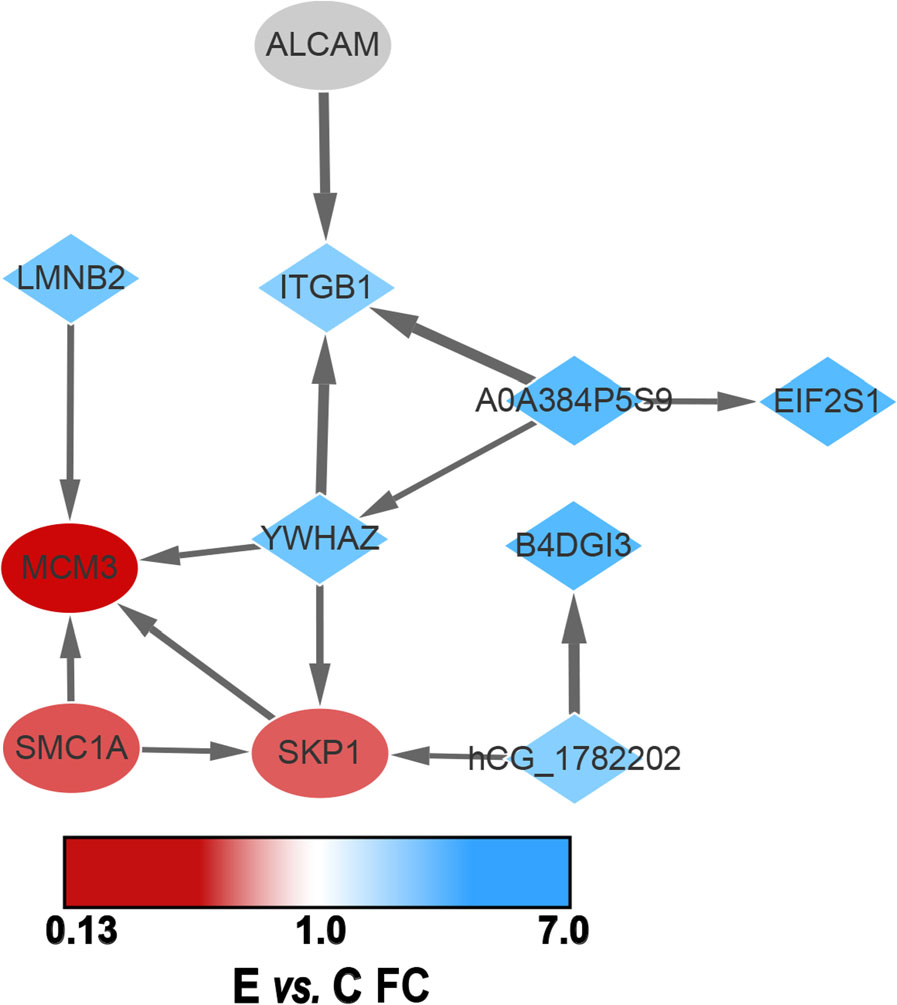
By PPI analysis of 11 DEPs involved in ‘Apoptosis’, ‘Cell adhesion molecules’, and ‘Cell cycle’ pathway. Down-regulated proteins (ellipse) and up-regulated proteins (diamond) were named by gene/protein name and the width of the arrow is related to the relationship score between these proteins

Enriched KEGG analysis revealed some significant pathways: ‘Apoptosis’ (5 proteins), ‘Cell adhesion molecules’ (2 proteins), and ‘Cell cycle’ (4 proteins), these results suggested that the occurrence of AUB-E was the result of multiple pathway dysfunction (as Supplementary Table S4). By PPI analysis, 11 DEPs (MCM3, SMC1A, SKP1, ALCAM, TNF receptor-associated factor, EIF2S1, epididymis secretory sperm binding protein, YWHAZ, LMNB2, hCG_1782202, and ITGB1) interacted with each other and enriched into a larger protein interaction network (Fig. 4), including 4 down-regulated proteins (ellipse) and 7 up-regulated proteins (diamond). Among up-regulated proteins, TNF receptor-associated factor and epididymis secretory sperm binding protein both retrieved no corresponding gene from the Universal Protein Resource (https://www.uniprot.org/, UniProt) and hCG_1782202 (E/C ratio: 1.79) which proteins do not have related commercial antigen. Meanwhile, EIF2S1(E/C ratio: 2.23), YWHAZ (E/C ratio: 1.99), LMNB2 (E/C ratio: 1.97), ITGB1 (E/C ratio: 1.77) were up-regulated. Moreover, a down-regulated protein, ALCAM (*P* = 6.71) did not be almost expressed in group E without an E/C ratio. Two down-regulated nucleus proteins: SKP1 (E/C ratio: 0.34) and MCM3 (E/C ratio: 0.15) were involved in the cell cycle pathway and have been linked with SMC1A (E/C ratio: 0.32) which was the only differentially expressed protein validated by a later study.

### Validation of the biomarker by western blot and immunohistochemistry

**Fig. 5:**
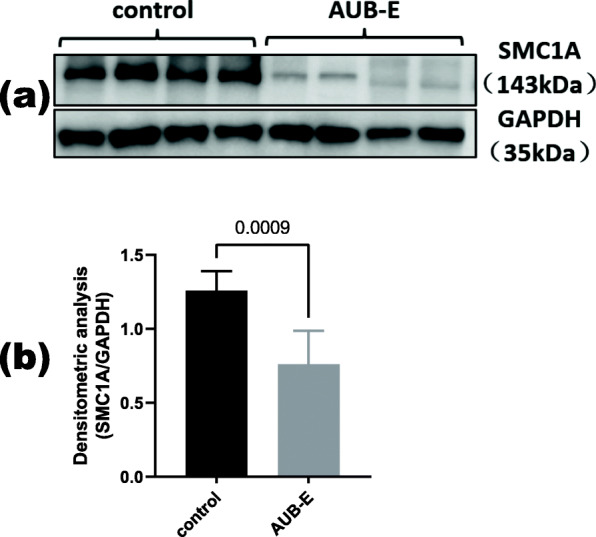
Western blot analysis showing SMC1A protein from control and AUB-E subjects. (a) Western blot analysis of SMC1A protein. GAPDH was used as a loading control. (b) Quantification of the expression of SMC1A proteins shows significantly decreased levels in the AUB-E group. Data are presented as mean ± SD. The *t*-test was used

**Fig. 6:**
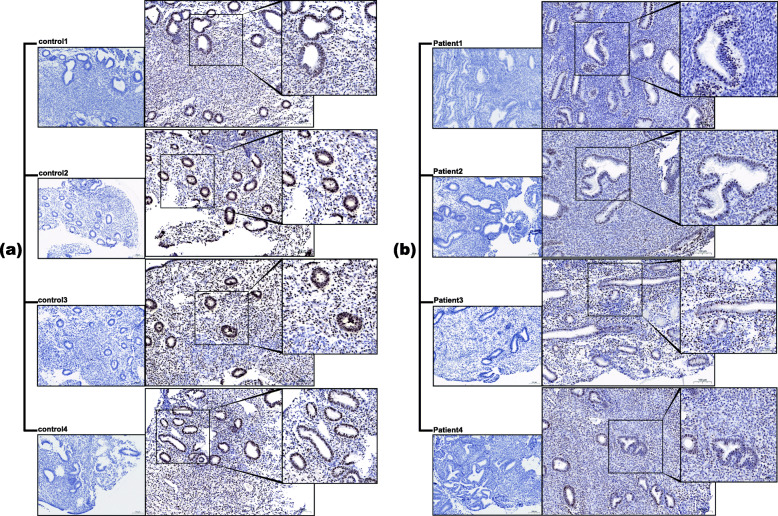
Immunohistochemical staining of SMC1A protein in human endometrium. (a) for group C; (b) for group E. Negative control was shown in the lower-left rectangles. (original magnification × 100)

**Fig. 7:**
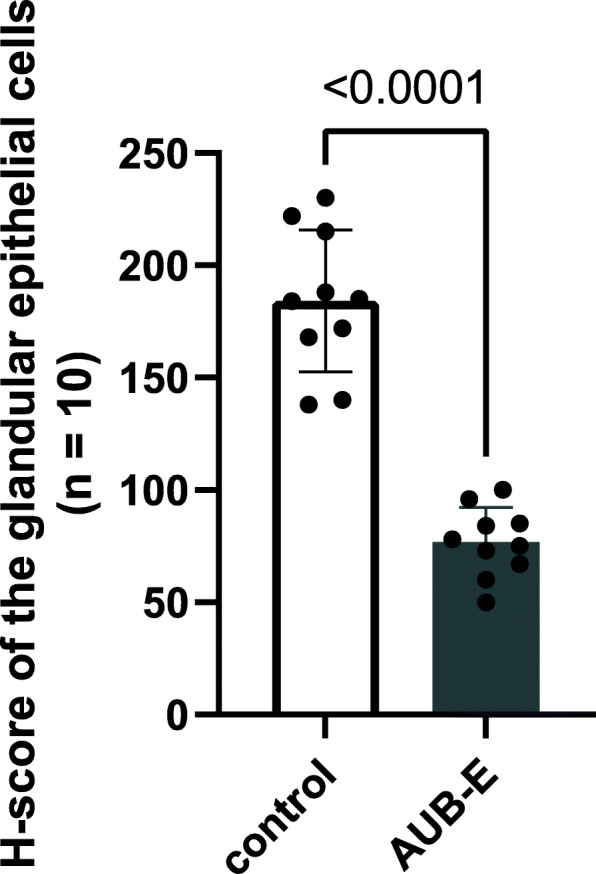
H-score of SMC1A expression in the glandular epithelial cells between the two groups (*n* = 10). The *t*-test was used

For validating purpose protein, SMC1A protein was analyzed by western blotting in the above samples (Fig. 5). Comparing the SMC1A expression levels between the two groups, SMC1A is low expressed in group E (*P* = 0.0009) following the same expression pattern observed in the proteomic analysis. Moreover, the other 10 patients in each group were retrieved through the electronic medical system following the above classification criteria (Table S5). Immunohistochemical staining of SMC1A was mainly expressed in glandular epithelial cells of the endometrium and partly in the stroma area (Fig. 6). Besides, H-scores of SMC1A expression in the glandular epithelial cells were similarly subject to significant decreases in the AUB-E group (184 ± 31.60 vs. 77 ± 15.43, *P*< 0.0001), but no significant in endometrial stromal cells (see Fig. 7).

## Discussion

While AUB is a prevalent symptom among women seeking gynecologic care, endometrial function in the context of menstruation and its disorders is still not fully understood [17]. For the present, the diagnosis of AUB-E depends on careful history taking and exclusion of other contributors [4, 18]. AUB-E is thought to be caused by a local disturbance in endometrial function-deficiencies or excesses of proteins or other entities that have an adverse effect on hemostasis, normal angiogenesis, vascular integrity, or endometrial repair [2, 4]. Nevertheless, the contributions to AUB-E are not completely understood, especially after the “PALM-COEIN” classification system was applied. In this study, we have focused on proteomic analysis, as a powerful tool to identify proteins possibly involved in pathogenesis, with the hypothesis that functionally important protein changes, will identify biomarkers of relevance to endometrial disorder (AUB-E).

In clinical practice, the diagnosis, evaluation, and treatment of HMB are based upon “patient experience”, the woman’s assessment of her blood loss and its impact on her life [8, 19, 20]. Therefore, the PBAC combined with self-reported to quality-of-life issues for women with excessive blood loss were used to assess the volume of menstrual blood loss in our study. Although Hb and HCT levels were not significant differences between the two groups, 2 patients presented anemia and all subjects in group E complained of a situation of HMB with a significant difference of PBAC scores in Table 2 and Supplementary Table S5.

Previous studies indicated that if patients performed a symptom of HMB, a primary disorder of mechanisms regulating local endometrial “hemostasis” itself may exist. Other primary endometrial disorders may not manifest in HMB but cause intermenstrual or prolonged bleeding. Prolonged bleeding may be a manifestation of deficiencies in the molecular mechanisms of endometrial repair [18]. It should be noted that AUB-related researches published in the past generally focused on the previous term “dysfunctional uterine bleeding” that could be confused with other causes of AUB [2]. Therefore, the results of these studies were limited. In this study, all participants in group E were excluded from other contributors of AUB, and self-reported predictable and cyclical ovulatory cycles with a situation of HMB. PCA analysis was used to reflect the total difference between the two groups and the variability among each group. Notably, the two groups were well discriminated into two blocks in our study. Meanwhile, the protein-level volcano plots of log_2_ FC and the *P* value between the AUB-E and control groups were shown in Fig. 3 that was very similar to the normal distribution. These results suggested that this experimental procedure was performed without significant bias toward different samples.

SMC1A, a member of the SMC superfamily and core structural component of the cohesin complex, is essential for sister chromatid cohesion, DNA recombination and repair, cell cycle regulation, genomic stability maintenance, and tumorigenesis [21, 22]. However, there was no relative study about SMC1A expression in the human endometrium or any gynecological diseases, which limited the function of SMC1A in this disease. In this study, our results revealed that SMC1A expression of proliferative-phase endometrium in women with AUB-E was significantly lower than in healthy women. It may indicate a genesis of AUB-E that SMC1A affected the function of endometrial repair.

The endometrium is morphologically divided into functional and basal layers. During endometrial repair and proliferation, mitosis occurs in the functional layer of the endometrium, a highly active layer consisting of glands supported by stroma [23]. Immunohistochemical analysis in other 20 samples demonstrated a change of SMC1A expression mainly located in the glandular epithelial cells, which indicated disturbances to endometrial remodeling after menstruation may underpin AUB-E. Although lack of research on SMC1A in endometrial epithelial cells, SMC1A knockdown appeared to coincide with cell cycle arrest and/or increased apoptosis in human breast, lung, prostate, and colorectal cancer cells in previous reports [24, 25], which indicated SMC1A protein may affect cell proliferation and inhibit apoptosis regulated the cell cycle progression [26–28]. Here, enriched KEGG pathway analysis revealed that SMC1A was down-expressed in the AUB-E group which was involved in the cell cycle pathway. The dynamics of SMC1A during the proliferative phase may be closely associated with cell proliferation and/or increased apoptosis in glandular epithelial cells regulated local endometrial “hemostasis”, which caused HMB. Unfortunately, the interactive proteins with SMC1A protein in the PPI analysis were not confirmed in our study.

### Limits

The main limitation of this study was a small number of samples. It is thus essential that any proposed biomarkers are subject to analysis on sufficiently large sample cohorts from multiple sites. Additional studies are needed with molecular functional studies to demonstrate the physiologic roles of SMC1A and provide mechanistic insight.

## Conclusion

Given the prevalence of AUB, there is a clear need for understanding the genesis of AUB for offering the best results of individualized and personalized diagnosis and care. In this study, a label-free proteomic strategy for the first time was employed to compare the proteomics of human proliferative-phase endometrium between AUB-E and control groups to meet the urgent biomedical and research need. 291 DEPs were enriched KEGG analysis and revealed SMC1A down-regulated in the endometrial tissues involved in the cell cycle pathway. Further western blotting and immunohistochemical analysis validated down-regulation of SMC1A located in endometrial glandular epithelial cells in AUB-E patients may demonstrate that SMC1A potentially inhibited the epithelial cells proliferation during endometrial remodeling contributed to a situation of HMB. Although there were some limitations, this study provided up-to-date information on the cause of AUB and the potential mechanism of SMC1A in AUB-E patients that is imperative for the advancement of women’s health-related quality of life.

## Supplementary Information

**Additional file 1: Supplementary Table S1.** Quantified protein groups in all human endometrial samples.

**Additional file 2: Supplementary Table S2.** different proteins in human endometrium between AUB-E and control.

**Additional file 3: Supplementary Table S3.** DEPs in human endometrium with AUB-E vs. control.

**Additional file 4: Supplementary Table S4.** Enrichment KEGG pathway analysis of the DEPs.

**Additional file 5: Supplementary Table S5.** The clinical details of other retrieved subjects for IHC.

**Additional file 6: Supplementary Fig. S1.** Heat map of the DEPs between AUB-E and control.

## Data Availability

The dataset supporting the conclusions of this article is included within the article and its additional files.

## Abbreviations

AUB: Abnormal uterine bleeding
FIGO: International Federation of Gynecology and Obstetrics
MS: mass spectrometry.
PBAC: Pictorial blood assessment chart.
PBS: phosphate-buffered saline.
FASP: filter-aided sample preparation.
IAA: iodoacetamide.
CAN: acetonitrile.
TFA: formic acid.
HPLC: High-performance liquid chromatography.
LTQ: linear ion trap.
FDR: false discovery rate.
FC: fold change.
DEPs: differentially expressed proteins.
PCA: principal components analysis.
KEGG: Kyoto Encyclopedia of Genes and Genomes.
PPI: protein-protein interactions.
SMC1A: structural maintenance of chromosomes protein 1 A.
PVDF: polyvinylidene fluoride.
BSA: bovine serum albumin.
TBST: tris buffered saline with Tween 20.
SD: standard deviation.
H-score: histological score.
Hb: hematoglobin.
HCT: hematocrit
