# Supplementary material for: Label-free proteomics uncovers SMC1A expression is Down-regulated in AUB-E
Source: Reprod Biol Endocrinol. 2021 Mar 2;19:35. doi: 10.1186/s12958-021-00713-4 (PMC7923474; doi:10.1186/s12958-021-00713-4)
Supplement: Supplementary file 5 — Additional file 5: Supplementary Table S5. The clinical details of other retrieved subjects for IHC. [file 12958_2021_713_MOESM5_ESM.pdf]

Table S5. The clinical details of the other 20 subjects for IHC.

Clinical characteristic	AUB-E<br>n = 10	Control<br>n = 10	*P* value
**Age, years**<br>Mean (SD)	43 (6)	39 (6)	0.239[b]
**Menarche, years**<br>Median (P_{25}, P_{75})	14 (13, 14)	14 (13, 14)	0.489[a]
**Body-mass index, kg/m²**<br>Mean (SD)	21.48 (2.38)	21.68 (1.74)	0.828[b]
**Menstruation duration, days**<br>Mean (SD)	5.5 (1.4)	5.6 (1.4)	0.870[b]
**Menstrual cycle length, days**<br>Median (P_{25}, P_{75})	30 (29.5, 30)	30 (28, 31)	0.932[a]
**PBAC score**<br>Median (P_{25}, P_{75})	137 (120, 145)	68 (63.8, 74.3)	0.000[a,*]

n, the number of subjects.

[a]*P* value for the Mann–Whitney U-test. [b]*P* value for the *t*-test.

*: $P < 0.05$, which denoted a significant difference between control and AUB-E groups.
