# Supplementary figures and images for: Label-free proteomics uncovers SMC1A expression is Down-regulated in AUB-E
Source: Reprod Biol Endocrinol. 2021 Mar 2;19:35. doi: 10.1186/s12958-021-00713-4 (PMC7923474; doi:10.1186/s12958-021-00713-4)

E.vs.C

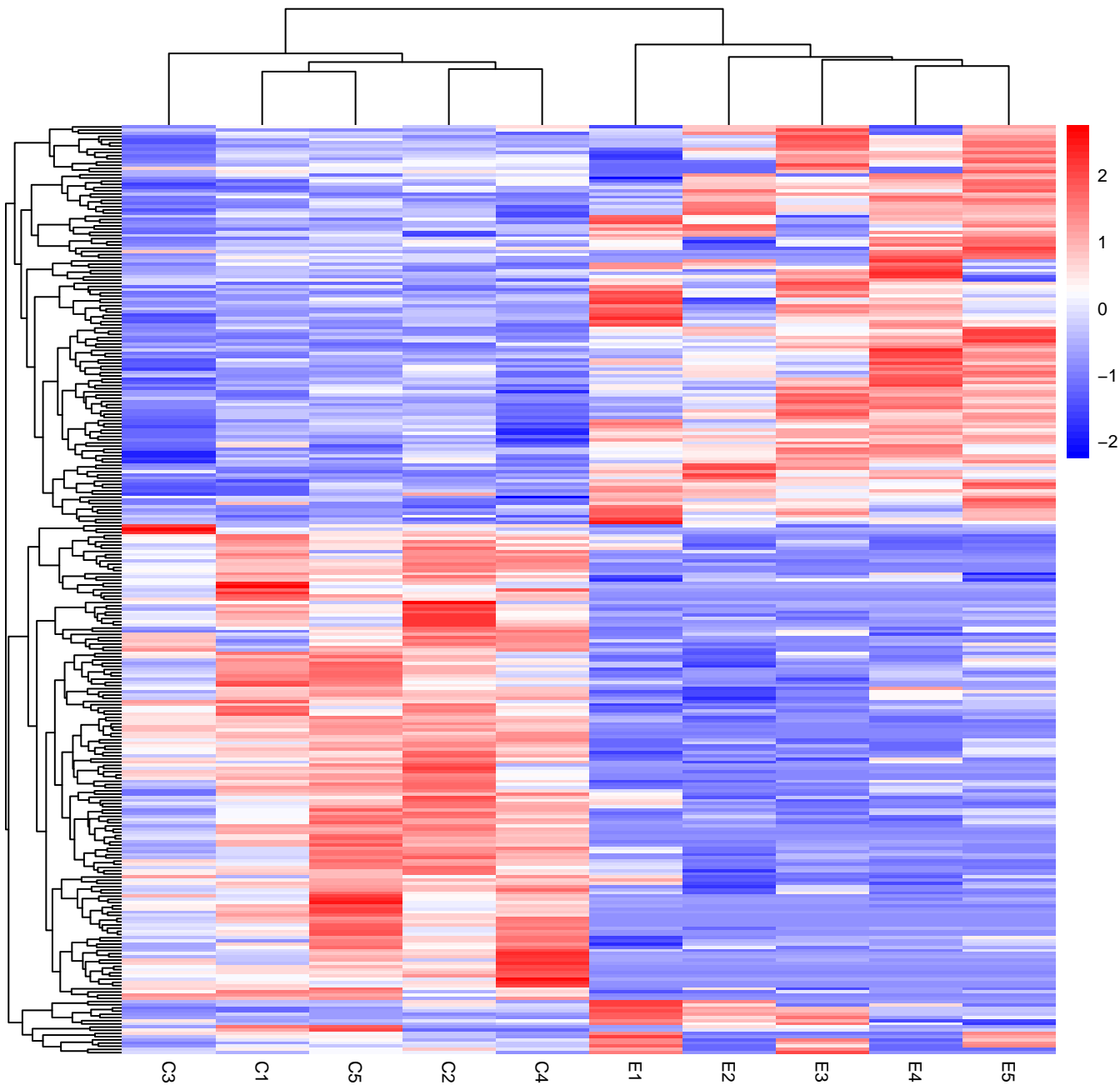

Supplement: Supplementary file 6 — Additional file 6: Supplementary Fig. S1. Heat map of the DEPs between AUB-E and control. [file 12958_2021_713_MOESM6_ESM.pdf]
